# Identification of Immunological Characteristics and Immune Subtypes Based on Single-Sample Gene Set Enrichment Analysis Algorithm in Lower-Grade Glioma

**DOI:** 10.3389/fgene.2022.894865

**Published:** 2022-05-13

**Authors:** Yunyang Zhu, Songwei Feng, Zhaoming Song, Zhong Wang, Gang Chen

**Affiliations:** ^1^ Department of Neurosurgery, The First Affiliated Hospital of Soochow University, Suzhou, China; ^2^ Department of Obstetrics and Gynaecology, Zhongda Hospital, School of Medicine, Southeast University, Nanjing, China; ^3^ Department of Neurosurgery and Brain and Nerve Research Laboratory, The First Affiliated Hospital of Soochow University, Suzhou, China

**Keywords:** lower-grade glioma, immunogenomics, immune clusters, glioma, tumor-immune microenvironment

## Abstract

Few breakthroughs have been achieved in the treatment of lower-grade glioma (LGG) in recent decades. Apart from the conventional pathological and histological classifications, subtypes based on immunogenomics would provide reference for individualized treatment and prognosis prediction. Our study identified four immunotypes of lower-grade glioma (clusters A, B, C, and D) by bioinformatics methods in TCGA-LGG and two CGGA datasets. Cluster A was an “immune-cold” phenotype with the lowest immune infiltration and longest survival expectation, whereas cluster D was an “immune-rich” subtype with the highest immune infiltration and poor survival expectation. The expression of immune checkpoints increased along with immune infiltration degrees among the clusters. It was notable that immune clusters correlated with a variety of clinical and immunogenomic factors such as age, WHO grades, IDH1/2 mutation, PTEN, EGFR, ATRX, and TP53 status. In addition, LGGs in cluster D were sensitive to cisplatin, gemcitabine, and immune checkpoint PD-1 inhibitors. RTK-RAS and TP53 pathways were affected in cluster D. Functional pathways such as cytokine–cytokine receptor interaction, antigen processing and presentation, cell adhesion molecules (CAMs), and ECM–receptor interaction were also enriched in cluster D. Hub genes were selected by the Matthews correlation coefficient (MCC) algorithm in the blue module of a gene co-expression network. Our studies might provide an immunogenomics subtyping reference for immunotherapy in LGG.

## Introduction

Lower-grade gliomas were previously regarded as World Health Organization (WHO) grade I and grade II gliomas in contrast to high-grade gliomas. Nowadays, the concept of diffuse lower-grade gliomas (LGGs), which refer to WHO grades II and III astrocytomas, oligodendrogliomas, and oligoastrocytomas (Eckel-Passow et al., 2015; Zeng et al., 2018), was applied to better define the slowly invasive and relatively indolent progression features. With a 10-year median survival, nearly 70% of LGG patients tend to gradually transform into high-grade glioma patients in which the tumor immune microenvironment (TIM) and genetic changes play vital roles (Appolloni et al., 2019; Nejo et al., 2019). The prognosis of LGG could hardly be predicted accurately by conventional pathological and histological classifications; thus, new subtypes based on immunogenomics are urgently required.

Classifications based on molecular and genetic markers had been adapted since the 2016 WHO classification of the central nervous system tumors (Louis et al., 2016) and were emphasized in the 2021 version (Louis et al., 2021). Isocitrate dehydrogenase (IDH) mutation plays an important role in altering the tumor immune microenvironment. The decrease of PD-L1 in IDH mutation gliomas means a strong T-cell activation (Han et al., 2020). In LGG, oligodendroglioma and astrocytoma were further classified based on 1p/19q co-deletion, p53 mutation, alpha-thalassemia/intellectual disability syndrome X-linked (ATRX) mutation, and telomerase reverse transcriptase (TERT) promoter mutation in IDH1/2-mutant gliomas (Ohba et al., 2020). The tumor microenvironment (TME) of glioma could be delineated by infiltrating immunocytes and genetic landscapes. It has been reported that the innate immune cells would be manipulated and reprogrammed in the TME to facilitate the tumorigenesis, progression, and spread and subsequently lead to tumor immune evasion in gliomas (Zindl and Chaplin, 2010; Hinshaw and Shevde, 2019). Tumor mutational burden (TMB), which is closely correlated with immune infiltration, consists of the base substitutions, insertion, or deletion mutations of the whole exome. A study had classified LGG patients into two groups based on TMB and found that the infiltration of B lineage, CD4 T cells, CD8 T cells, neutrophils, macrophages, and dendritic cells would lead to shorter overall survival along with the high expression of immune checkpoints PD-1 and CTLA-4 (Yin et al., 2020).

Immune checkpoint inhibitors had promising therapeutic effects in several tumors (Lipson et al., 2015; Emens et al., 2017). The inhibition of PD-1 and CTLA-4 could notably enhance immunosurveillance and prolong the survival time in mouse glioma models (Wainwright et al., 2014; Xu et al., 2020). However, the clinical application remains challenging because of the “cold phenotype” of glioma (Qi et al., 2020). Our study would seek for the most suitable immunotyping for immune checkpoint blocking therapy.

Here, four immune clusters of LGG based on The Cancer Genome Atlas (TCGA) immune datasets were identified and then verified in two Chinese Glioma Genome Atlas (CGGA) datasets. The immune cell infiltration features, hub genes, potential drugs, and prognosis were studied by bioinformatics methods. This study might offer reference for immunogenomics subtyping for individualized LGG therapy.

## Methods

### Data Processing

RNA-seq data (level-3, HTseq-FPKM) and clinical information of lower-grade glioma (LGG, grade II–III) samples were obtained from The Cancer Genome Atlas (TCGA) dataset. A total of 481 samples were finally selected after removing samples with no survival state, no WHO grade, recurrent tumor, reduplicated sequencing, and whose survival time were less than 1 day. In addition, RNA-seq and clinicopathological data were obtained from the Chinese Glioma Genome Atlas (CGGA) website as the validation set. A total of 332 samples with complete survival information were chosen from the CGGA-693 dataset (CGGA-LGG-1) and 162 samples were obtained from the CGGA-325 dataset (CGGA-LGG-2). The batch effect correction was performed by the R package termed “SVA.”

### Identification of Immune-Related Clusters in Lower-Grade Gliomas

Single-sample Gene Set Enrichment Analysis (ssGSEA) was conducted in the three datasets based on the expression level of 29 immunity-associated signatures by the R package “GSVA.” Consensus clustering was then applied to define the immune subgroups based on the ssGSEA scores by the “ConsensusClusterPlus” package in R. The K-means clustering algorithm was performed with 100 resampling iterations by random selection of 80% of the total samples to ensure the clustering stability. The best cluster number was determined by the consensus matrix (CM) heat maps, cumulative distribution function (CDF) curves, and delta area score of CDF curves. A principal component analysis (PCA) was used to illustrate the reliability of optimal methods. Thorsson et al. (2018) had identified six immune function subtypes by an extensive immunogenomic analysis: wound healing (C1), IFN-γ dominant (C2), inflammatory (C3), lymphocyte depleted (C4), immunologically quiet (C5), and TGF-β response (C6). A Sankey plot was applied to visualize the relationships between our four clusters and the six identified immune functional subtypes mentioned previously.

### Features of Immune Cell Clusters in the Immune Microenvironment of Lower-Grade Gliomas

The Estimation of Stromal and Immune cells in Tumors using Expression data (ESTIMATE) algorithm was used to evaluate the LGG microenvironment (Yoshihara et al., 2013). Immune scores and stromal scores were calculated to reveal the abundance of infiltrating immune and stromal cells. ESTIMATE scores were calculated for reflecting non-tumor composites. Tumor purity was inferred by the aforementioned scores. The Kruskal–Wallis test was used to compare differences in multiple clusters. Heat maps were drawn by the “pheatmap” package in R.

### Estimation of Immune Cell Infiltration

The Microenvironment Cell Populations-counter (MCP counter) algorithm (Becht et al., 2016) was used to quantitate the abundance of immunocytes in heterogeneous tissues by the “MCPcounter” R package. The absolute abundances of two stromal cells and eight immune cells were evaluated by immune cell scores, including T cells, CD8 T cells, cytotoxic lymphocytes, B lineage, NK cells, monocytic lineage, myeloid dendritic cells, neutrophils, endothelial cells, and fibroblasts.

### Prediction of Potential Drugs

The SubMap analysis (Hoshida et al., 2007; Roh and Chen, 2017) from Gene Pattern (https://www.genepattern.org/) was used to predict the response to immune checkpoint blockade (anti-PD-1 and anti-CTLA-4 immunotherapy). In addition, the chemotherapy response was predicted based on the public pharmacogenomics database termed “Genomics of Drug Sensitivity in Cancer” (GDSC, http://www.cancerrxgene.org). The half-maximum inhibitory concentration (IC50) of each patient was estimated by the R package “pRRophetic” with Ridge’s regression, and the accuracy of the prediction was estimated by a 10-fold cross-validation. The IC50 of each sample in TCGA dataset was calculated based on the prediction models of bleomycin and doxorubicin, and cisplatin and gemcitabine.

### Gene Set Enrichment Analysis

The GSEA algorithm was used to investigate the biological functions and pathways of clusters A and D, with C2:CP KEGG gene sets from MSigDB as the reference gene sets. False discovery rate (FDR) < 0.05 was the screening threshold.

### Weighted Gene Co-Expression Networks Analysis and Protein–Protein Interaction Networks Analysis

A weighted gene co-expression networks analysis (WGCNA) algorithm was used to mine the synergistically expressed gene modules. Immune-related genes from the ImmPort dataset (https://www.immport.org/) were classified into different modules which were significantly correlated with the four immune clusters by the R package “WGCNA.” Samples were clustered by a hierarchical clustering algorithm implemented in the R function “hclust.” The soft thresholding power β = 3 was selected by the R function “pickSoftThreshold” (scale free R^2^ = 0.85). The expression matrix was converted into the adjacent matrix and then into the topological matrix for gene clustering. An average linkage hierarchical cluster approach was used to cluster genes into a dendrogram. The STRING database (Szklarczyk et al., 2011) was explored to construct the protein–protein interaction (PPI) network. In the PPI gene network of the target module, hub genes were the top ten genes ranked by the MCC algorithm of “cytoHubba” plugin in Cytoscape 3.8.0. In addition, the survival curves based on the best cut-off value of hub genes were drawn by the “survminer” package in R.

### Statistical Analysis

Student’s t-test was applied for normal distributions, and the Mann–Whitney U-test was performed for nonparametric distribution. Chi-square or Fisher’s exact tests were used for categorical data. Kaplan–Meier curves and log-rank tests were used to evaluate the survival time of different immune clusters. The nonparametric Kruskal–Wallis (KW) test was used to analyze the difference in IC50 in different clusters. The Benjamini–Hochberg procedure was applied to control the false discovery rate (FDR) for multiple testing. *p* < 0.05 was considered statistically significant (* represented *p* < 0.05, ** referred *p* < 0.01, and *** referred *p* < 0.001).

## Results

### Identification of Four Immune-Related Clusters in Lower-Grade Gliomas

**FIGURE 1:**
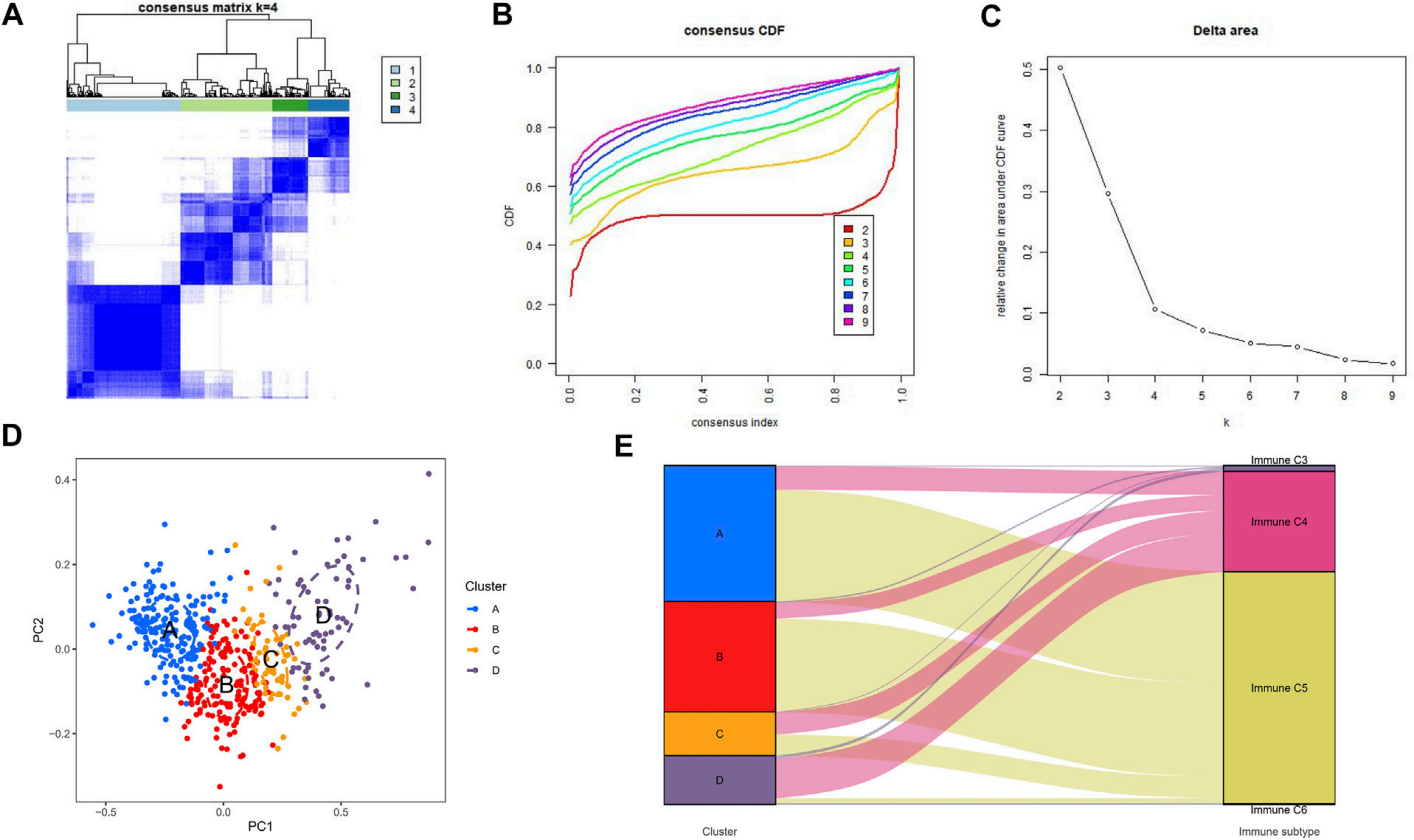
Consensus clustering results in TCGA-LGG cohorts. **(A)** Consensus clustering matrix of *k* = 4 as the optimal cluster number. **(B)** CDF curves of the consensus score from *k* = 2–9. **(C)** Delta area under the CDF curve. **(D)** Principal component analysis for the ssGSEA scores of four clusters. Each subgroup was distinguished by different colors. **(E)** Sankey diagram to visualize the relationships between our four clusters and the six identified immune function subtypes.

Unsupervised consensus clustering was applied to explore a novel immune classification of LGGs based on the ssGSEA scores of TCGA dataset. The optimal clusters number was found to be four with maximal consensus within clusters and minimal ambiguity among clusters (Figures 1A–C). PCA verified that the ssGSEA scores could completely be distinguished into four subtypes which were referred to as cluster A, cluster B, cluster C, and cluster D in TCGA dataset (Figure 1D). The clustering results were the same in CGGA-1 (Supplementary Figure S1A) and CGGA-2 datasets (Supplementary Figure S1B). The Sankey diagram revealed the immune function characteristics of the four clusters (Figure 1E). The majority of clusters A and B were related to the C5 function of “Immunologically Quiet.” Cluster D was related to the C4 function of “Lymphocyte Depleted.”

### Landscape of the Four Clusters in the Tumor Immune Environment in Lower-Grade Gliomas

**FIGURE 2:**
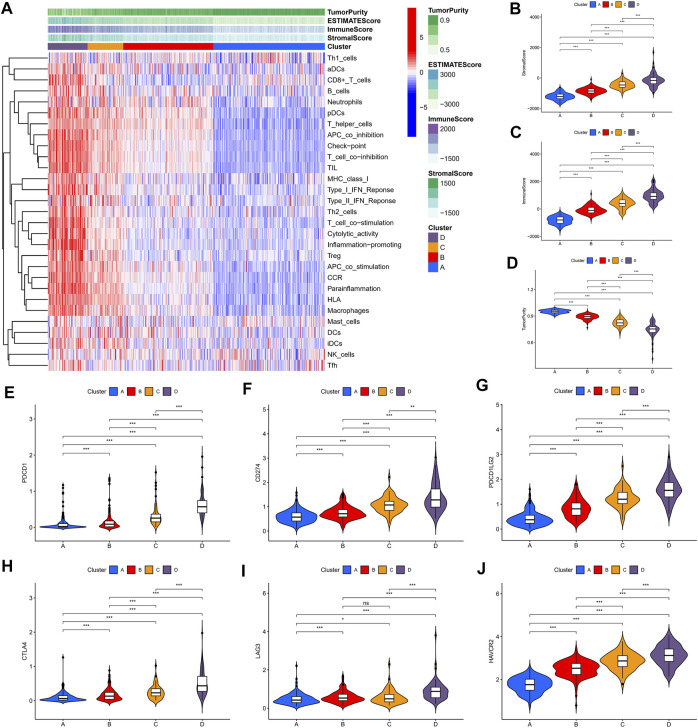
Immune characteristics of the four clusters in the TCGA dataset. **(A)** Heat map of the four immune clusters based on ssGSEA scores. **(B)** Stromal scores, **(C)** immune scores, and **(D)** tumor purity of different clusters. **(E–J)** Violin plots for the immune checkpoint gene expressions of PDCD1, CD274, PDCD1LG2, CTLA-4, LAG3, and HAVCR2 in different clusters.

**FIGURE 3:**
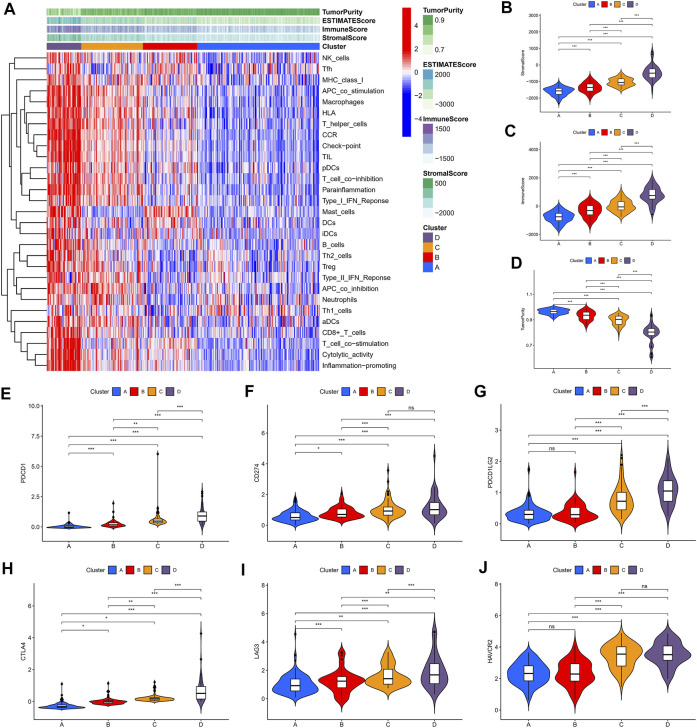
Immune characteristics of the four clusters in the CGGA-1 dataset. **(A)** Heat map of the four immune clusters based on ssGSEA scores. **(B)** Stromal scores, **(C)** immune scores, and **(D)** tumor purity of different clusters. **(E–J)** Violin plots for the immune checkpoint gene expressions of PDCD1, CD274, PDCD1LG2, CTLA-4, LAG3, and HAVCR2 in different clusters.

**FIGURE 4:**
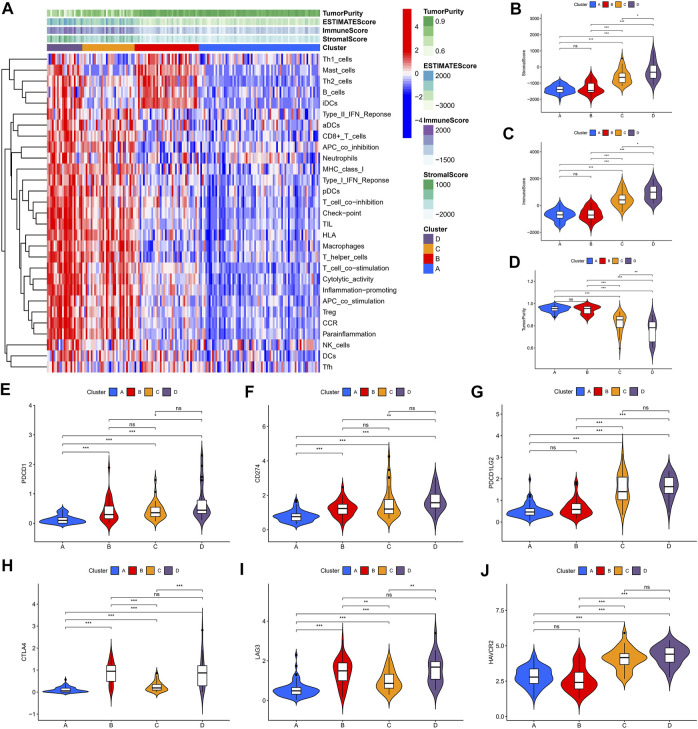
Immune characteristics of the four clusters in the CGGA-2 dataset. **(A)** Heat map of the four immune clusters based on ssGSEA scores. **(B)** Stromal scores, **(C)** immune scores, and **(D)** tumor purity of different clusters. **(E–J)** Violin plots for the immune checkpoint gene expressions of PDCD1, CD274, PDCD1LG2, CTLA-4, LAG3, and HAVCR2 in different clusters.

We identified four immune clusters and their immune characteristics in TCGA, CGGA-1, and CGGA-2 datasets (Figures 2A, 3A, 4A) were shown in the heat maps. Cluster D showed the highest degree of immune infiltration and it was followed by clusters C, B, and A. Cluster D was considered the “immune-rich” phenotype with the highest enrichment scores and the least tumor purity while cluster A was the opposite which was regarded as an “immune cold” phenotype (Figures 2A–D, Figures 3A–D, Figures 4A–D). Apart from that, clusters A and B could be seen as a “low-immune infiltration” subgroup whereas clusters C and D were considered as a “high-immune infiltration” subgroup. The expression of the immune checkpoint genes PDCD1 (PD-1), CD274 (PD-L1), PDCD1LG2 (PD-L-2), CTLA-4, HAVCR2, and LAG3 which played a vital role in the oncogenesis and progression of LGG were expressed in the following order: D > C > B > A (Figures 2E–J, Figures 3E–J, Figures 4E–J).

### Clinical Features and Gene Mutation Characteristics of the Four Immune Clusters

**FIGURE 5:**
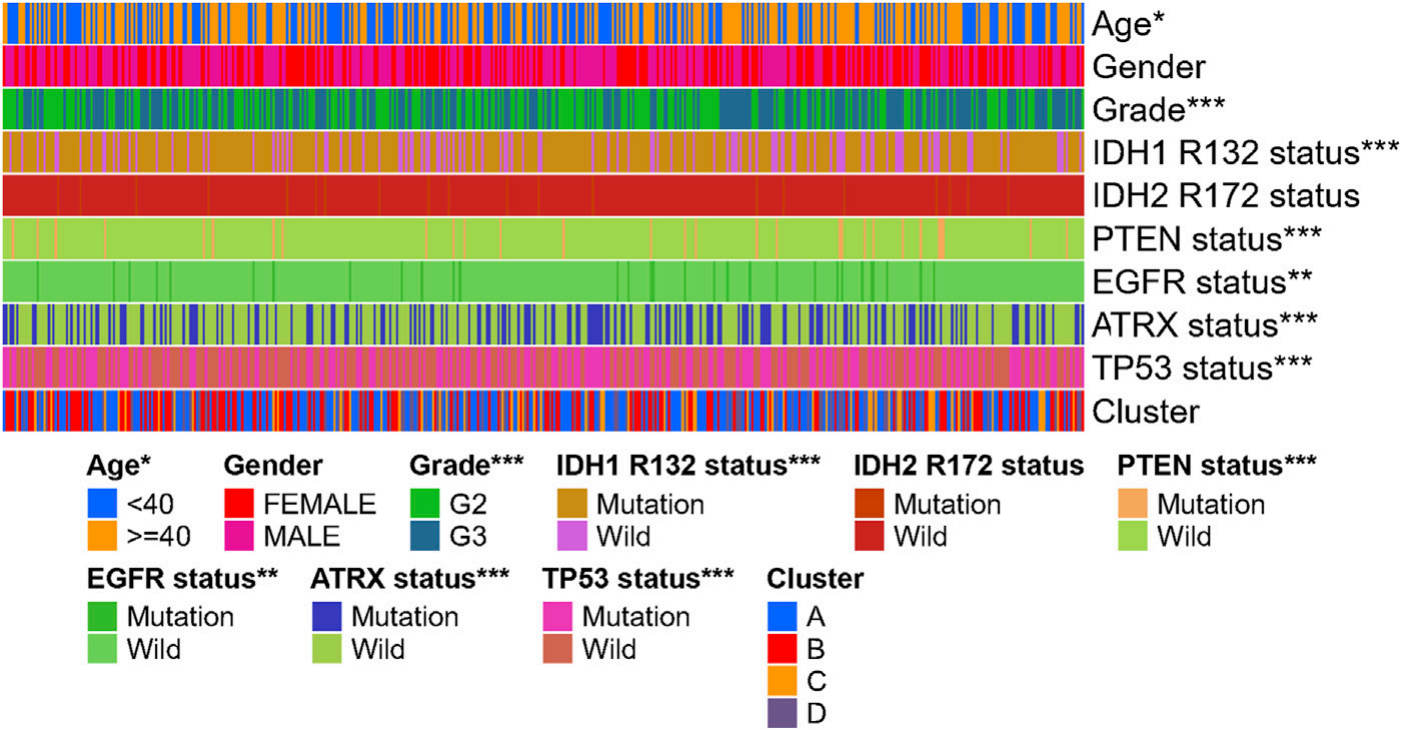
Heat map of clinical features and gene mutation. Characteristics of the four immune subtypes in the TCGA-LGG dataset.

**FIGURE 6:**
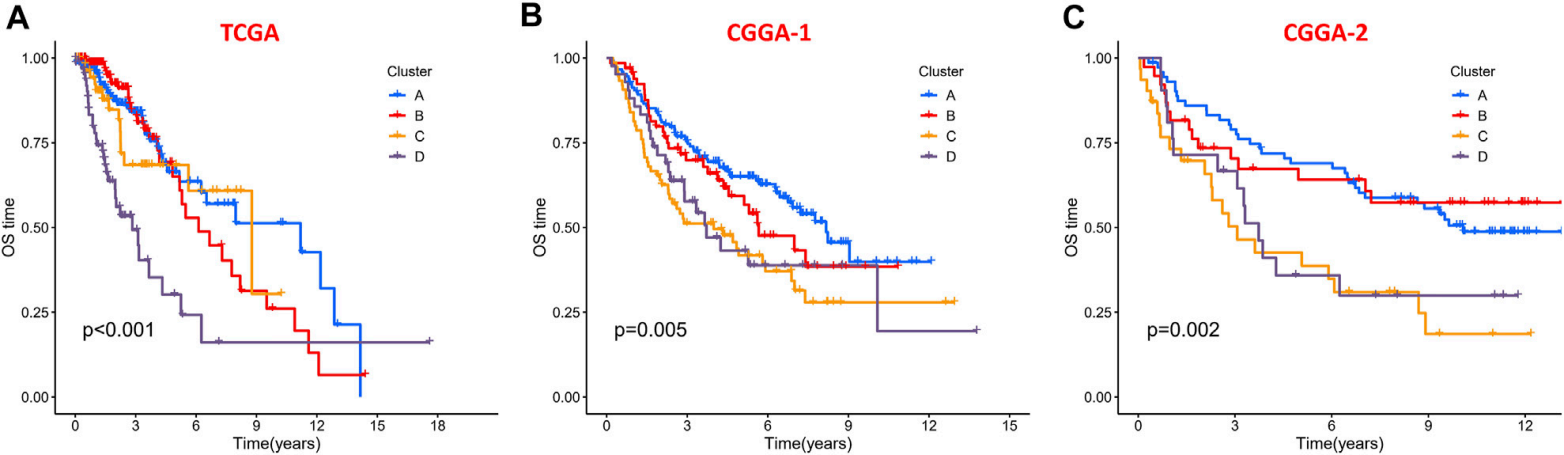
Kaplan–Meier survival curves of the four clusters in the **(A)** TCGA dataset, **(B)** CGGA-1 datasets, and **(C)** CGGA-2 datasets.

To evaluate the clinical features and gene mutation characteristics among the four immune clusters, age, gender, tumor grade, IDH1 (R132) status, IDH2 R172 status, PTEN status, EGFR status, ATRX status ,and TP53 status in TCGA dataset were counted (Table 1; Figure 5). In clusters A, C, and D, people aged more than 40 years accounted for more proportion, whereas those younger than 40 years were more common in cluster B. WHO grade II glioma tended to be common in the “low-immune infiltration” subgroup (clusters A and B), whereas the “high-immune infiltration” subgroup (clusters C and D) counted more in WHO grade III glioma. The frequency of IDH1 (R132) mutation was much higher in the “low-immune infiltration” subgroup than the “high-immune infiltration” subgroup. The frequency of PTEN and EGFR mutations was significantly higher in cluster D, which had the highest immune infiltration. In clusters B and C, which had mild immune infiltration changes, ATRX mutation frequencies were higher than those in clusters with extreme immune infiltration changes (clusters A and D). TP53 mutation was common in cluster B. Gender and IDH2 (R172) mutation status were not significant covariates in the immune classification. In addition, the “low-immune infiltration” subgroup (clusters A and B) showed longer overall survival than the “high-immune infiltration” subgroup (clusters C and D) (Figure 6), indicating that immune infiltration of LGG played a negative role in the prognosis.

**TABLE 1:** Correlations among the four immune clusters and clinical characteristics in the TCGA-LGG dataset.

Covariates	Cluster	Total	A	B	C	D	*p* value
Age	<40	221 (45.95%)	84 (43.3%)	85 (54.49%)	28 (45.16%)	24 (34.78%)	0.035
	≥40	260 (54.05%)	110 (56.7%)	71 (45.51%)	34 (54.84%)	45 (65.22%)	
Gender	FEMALE	214 (44.49%)	95 (48.97%)	60 (38.46%)	28 (45.16%)	31 (44.93%)	0.2738
	MALE	267 (55.51%)	99 (51.03%)	96 (61.54%)	34 (54.84%)	38 (55.07%)	
Grade	G2	230 (47.82%)	107 (55.15%)	83 (53.21%)	28 (45.16%)	12 (17.39%)	0
	G3	251 (52.18%)	87 (44.85%)	73 (46.79%)	34 (54.84%)	57 (82.61%)	
IDH1 R132 status	Mutation	368 (76.51%)	161 (82.99%)	135 (86.54%)	37 (59.68%)	35 (50.72%)	0
	Wild	113 (23.49%)	33 (17.01%)	21 (13.46%)	25 (40.32%)	34 (49.28%)	
IDH2 R172 status	Mutation	20 (4.16%)	12 (6.19%)	6 (3.85%)	1 (1.61%)	1 (1.45%)	0.2292
	Wild	461 (95.84%)	182 (93.81%)	150 (96.15%)	61 (98.39%)	68 (98.55%)	
PTEN status	Mutation	29 (6.03%)	5 (2.58%)	8 (5.13%)	4 (6.45%)	12 (17.39%)	2.00E-04
	Wild	452 (93.97%)	189 (97.42%)	148 (94.87%)	58 (93.55%)	57 (82.61%)	
EGFR status	Mutation	30 (6.24%)	7 (3.61%)	6 (3.85%)	6 (9.68%)	11 (15.94%)	0.001
	Wild	451 (93.76%)	187 (96.39%)	150 (96.15%)	56 (90.32%)	58 (84.06%)	
ATRX status	Mutation	174 (36.17%)	45 (23.2%)	83 (53.21%)	26 (41.94%)	20 (28.99%)	0
	Wild	307 (63.83%)	149 (76.8%)	73 (46.79%)	36 (58.06%)	49 (71.01%)	
TP53 status	Mutation	216 (44.91%)	66 (34.02%)	92 (58.97%)	29 (46.77%)	29 (42.03%)	1.00E-04
	Wild	265 (55.09%)	128 (65.98%)	64 (41.03%)	33 (53.23%)	40 (57.97%)	

### Comparison of Immune and Stromal Cells Among the Four Clusters

**FIGURE 7:**
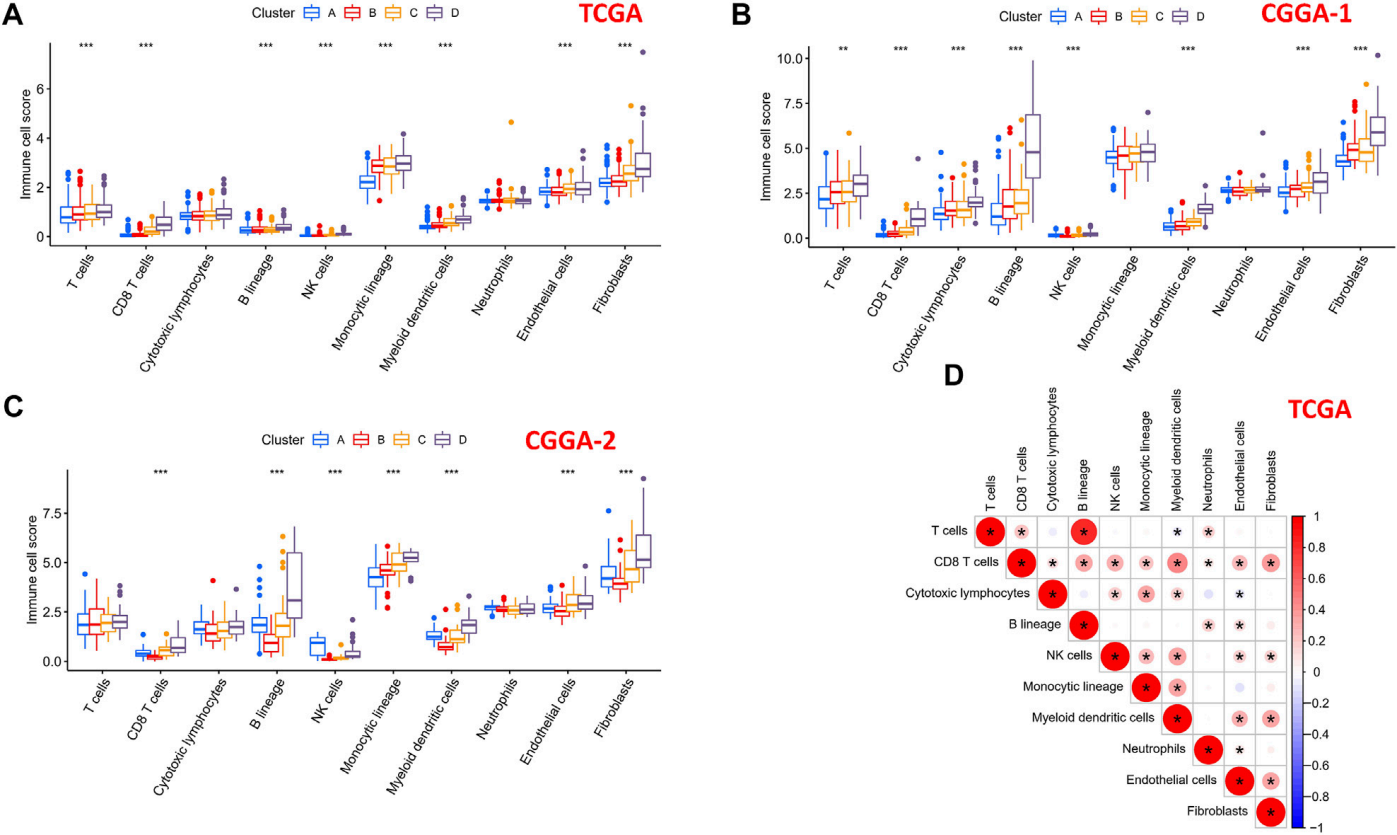
Immune cells scores of the four subtypes in 10 human immunocytes using the MCPcounter algorithm. **(A)** TCGA, **(B)** CGGA-1, and **(C)** CGGA-2 cohorts showed significantly different immune cell populations among the four subtypes. **(D)** Intrinsic correlation between infiltrating immunocytes and immune scores.

To explore the differential distribution of immunocytes and stromal cells in tumor immunity clusters, the MCPcounter algorithm was used to calculate the contents of two stromal cells and eight immune cells in the four clusters in TCGA, CGGA-1, and CGGA-2 datasets (Figures 7A–C). Immune cell scores of CD8 T cells, B lineage, NK cells, myeloid dendritic cells, endothelial cells, and fibroblasts in the “high-immune infiltration” subgroup (clusters C and D) were significantly higher than those in the “low-immune infiltration” subgroup (clusters A and B). Then, the correlation landscape of immunocytes was characterized to compare the relative subpopulations of infiltrating immune cells and immune scores among the four cluster patterns (Figure 7D). Cox regression analysis of the 10 immune cells in TCGA, CGGA-1, and CGGA-2 datasets are shown in Supplementary Figure S2, revealing the prognostic risk factors of infiltrating immunocytes.

### Therapeutic Potential of Different Immune Clusters

**FIGURE 8:**
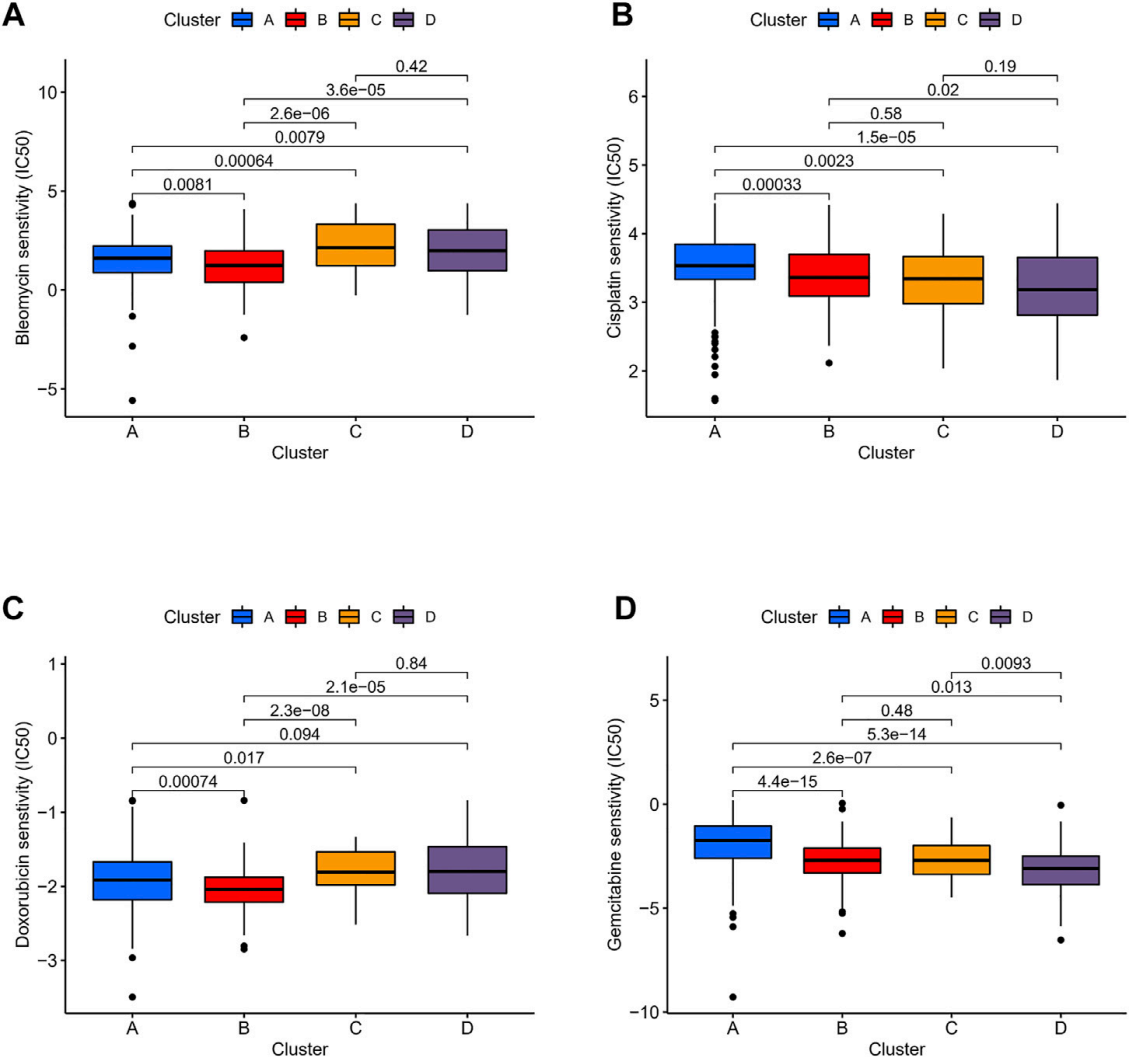
Sensitivity of chemotherapeutics in different immune clusters. Differences in IC50 of **(A)** bleomycin, **(B)** cisplatin, **(C)** doxorubicin, and **(D)** gemcitabine were estimated among the four immune clusters.

We compared the expression profiles of the four immune clusters in TCGA datasets by the Subclass Mapping algorithm which assessed the response to anti-PD-1 and anti-CTLA-4 therapies. A significant correlation was observed when comparing cluster D with the PD-1 response group (Bonferroni-corrected *p* = 0.001, Supplementary Figure S3A). It revealed that cluster D might have a better response to anti-PD-1 therapy while no significant correlation of anti-CTLA-4 therapy was observed in all the clusters. The “pRRophetic” algorithm was applied to evaluate the sensitivity of four common chemical drugs: cisplatin, bleomycin, doxorubicin, and gemcitabine for the four immune clusters. A lower IC50 value would indicate a better sensitivity in the prediction models. For bleomycin and doxorubicin, the “low-immune infiltration” subgroup (clusters A and B) was more sensitive than the “high-immune infiltration” subgroup (clusters C and D). For cisplatin and gemcitabine, cluster D was the most sensitive and cluster A was the least sensitive compared with the other clusters (Figures 8A–D). Moreover, to compare the accuracy of the four immune clusters, prognosis signatures in other references were used to compare the C-index. The results were also exciting: in the C-index for predicting the LGG survival possibility, our immune clusters showed better predictive value than other signatures (Maimaiti, Aierpati et al., 2022; Maimaiti, Aierpati et al., 2021) (0.813 > 0.774 > 0.712 > 0.662, Supplementary Figure S3B).

### Gene Set Enrichment Analysis

**FIGURE 9:**
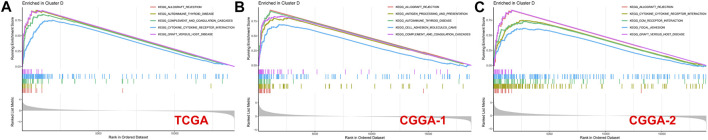
GSEA enrichment analysis for clusters A and D in **(A)** TCGA, **(B)** CGGA-1, and **(C)** CGGA-2 datasets. No significant enrichment was found in cluster A with a threshold of FDR <0.05.

A GSEA analysis was performed to screen the correlated biological pathways in immune clusters A and D. Cluster D was enriched in Allograft rejection, complement and coagulation cascades, cytokine–cytokine receptor interaction, graft versus host disease, antigen processing and presentation, cell adhesion molecules (CAMs), ECM-receptor interaction, and focal adhesion in TCGA, CGGA-1, and CGGA-2 datasets. Enrichment results of cluster A were not significant under the strict FDR <0.05 threshold in all the three datasets (Figure 9).

### Mutation Landscapes in Lower-Grade Gliomas

**FIGURE 10:**
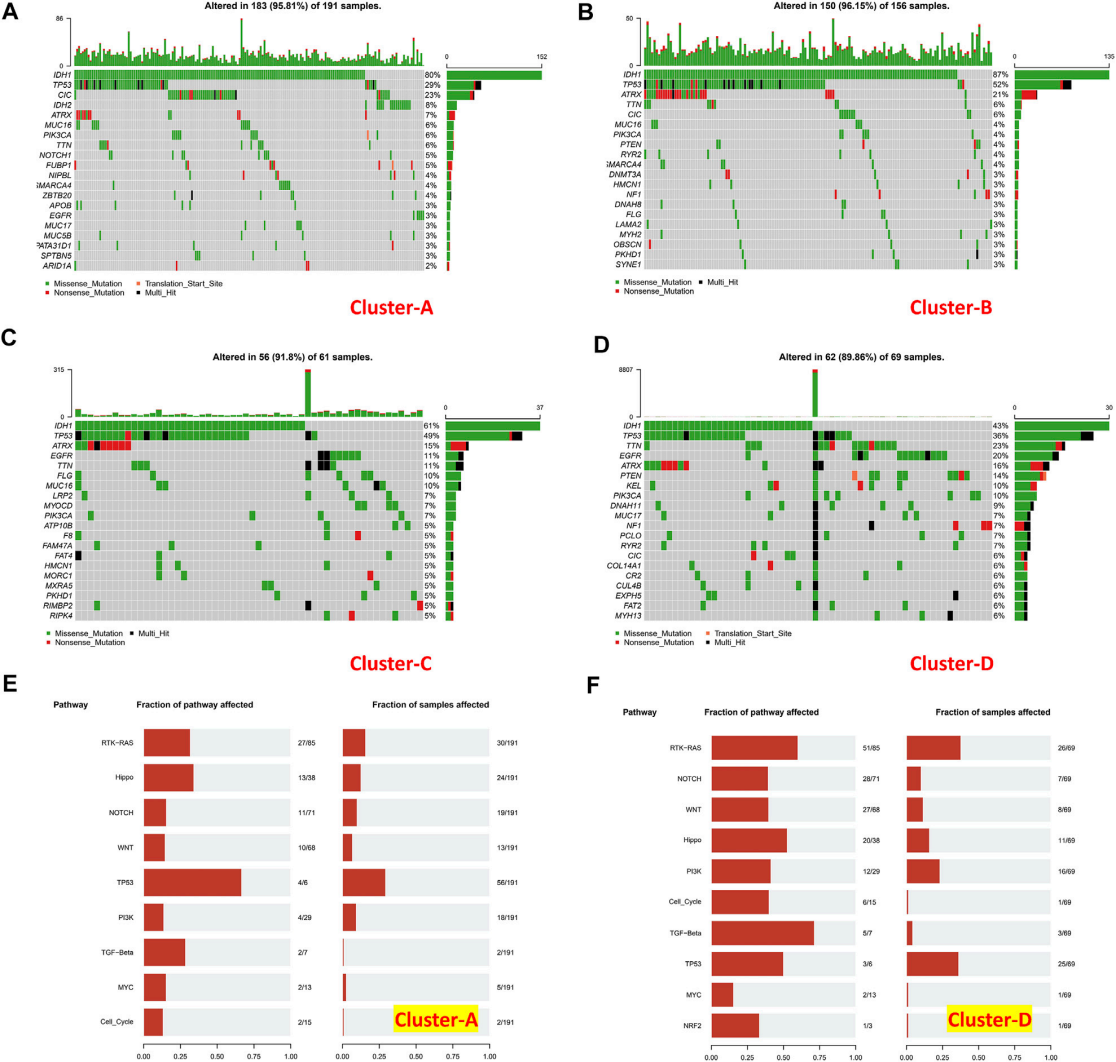
Mutation landscapes of immune clusters. **(A)** Gene mutation landscape in cluster A, **(B)** cluster B, **(C)** cluster C, and **(D)** cluster **D**. Pathways affected in **(E)** cluster A and **(F)** cluster **(D)**.

Tumor mutational burden of coding errors is reported to have a certain correlation with the tumor immune microenvironment. We explored this correlation of different immune clusters in TCGA-LGG datasets (Figures 10A–D). The frequency of IDH1 missense mutations in the “low-immune infiltration” subgroup was higher than that in the “high-immune infiltration” subgroup (80 and 87% in clusters A and B, 61 and 43% in clusters C and D). TP53 mutations were higher in clusters B (52%) and C (49%) than those in clusters A (29%) and D (36%). Meanwhile, most of them were missense mutations. The CIC missense mutation was high in cluster A. ATRX mutations including missense mutations, nonsense mutations, and multi-hit were at a high frequency in clusters B and C. TTN, EGFR, and ATRX mutations were common in the “high-immune infiltration” subgroup. PTEN, KEL, and PLK3CA mutations were higher in cluster D. the TP53 pathway was highly affected in cluster A and RTK-RAS and TP53 pathways were affected in cluster D (Figures 10E,F).

### Weighted Gene Co-Expression Network Analysis and the Immune Characteristics of Hub Genes

**FIGURE 11:**
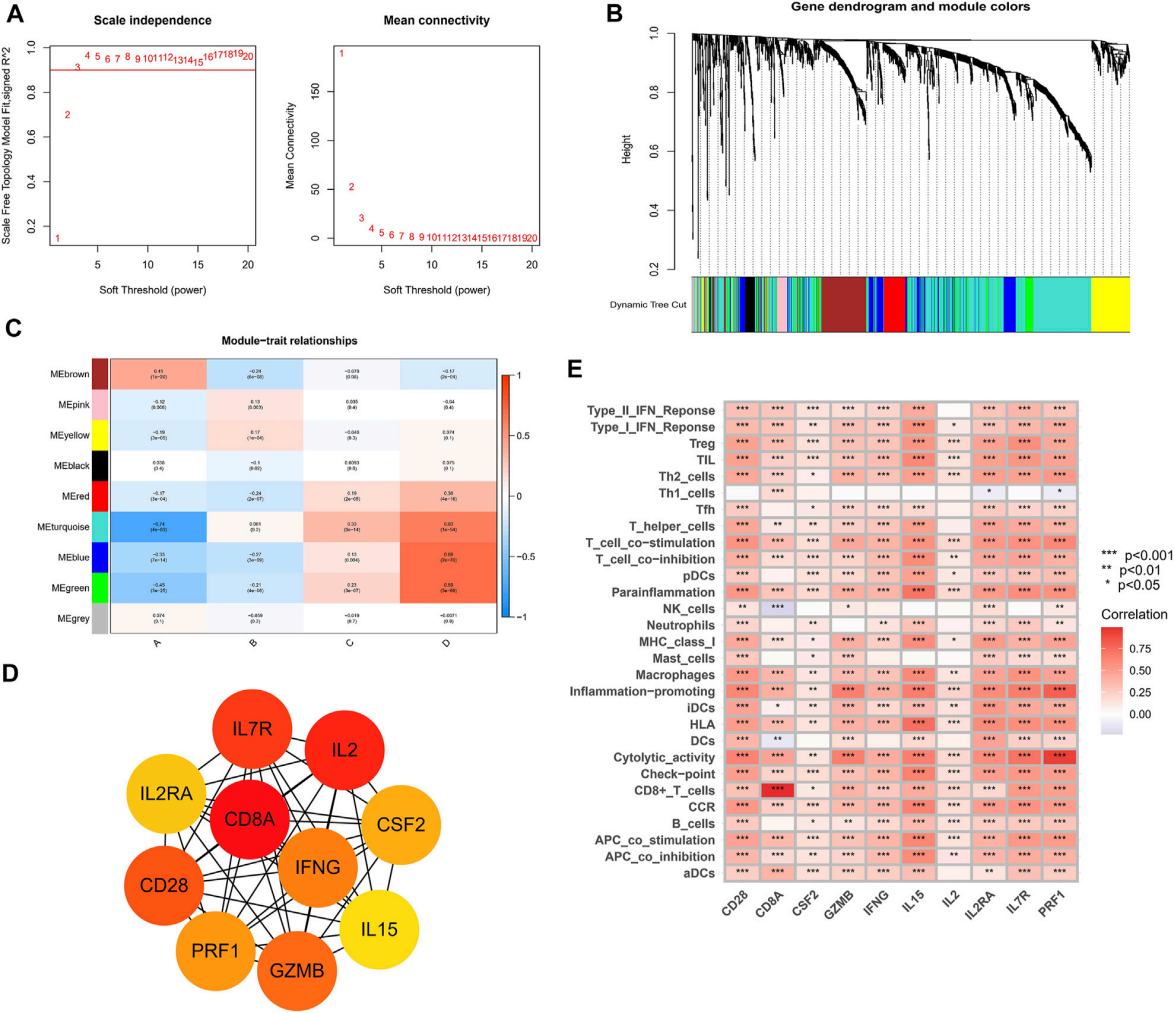
Weighed co-expression analysis of immuno-related genes in the TCGA cohort. **(A)** Selection of soft thresholding power **(B)** Gene dendrogram and the correlation modules. **(C)** Heat map of module-trait relationships. **(D)** Hub genes and their internal correlation network. **(E)** Correlation heat map for hub genes and immune gene sets.

**FIGURE 12:**
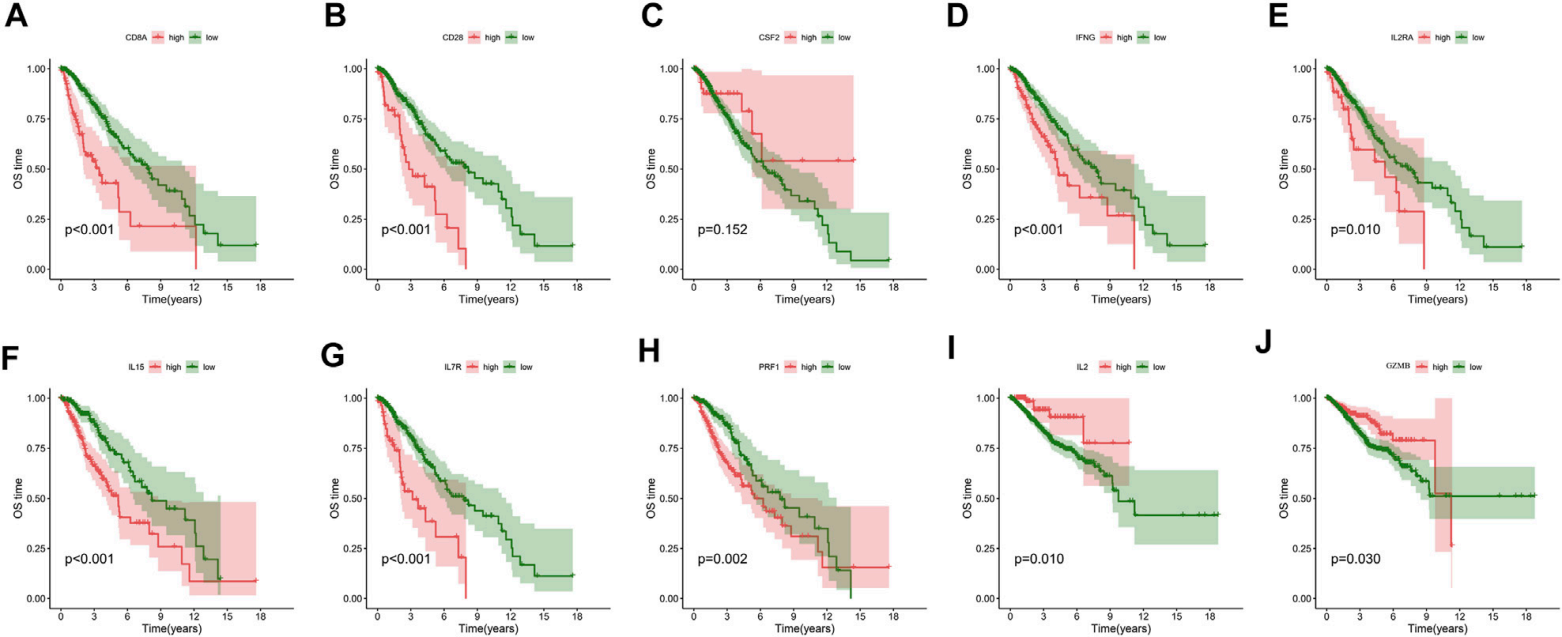
Overall survival analysis of **(A)** CD8A, **(B)** CD28, **(C)** CSF2, **(D)** IFNG, **(E)** IL2RA, **(F)** IL15, **(G)** IL7R, **(H)** PRF1, **(I)** IL2, and **(J)** GZMB.

The WGCNA networks of immuno-related genes with immune infiltrating clusters were constructed. The optimal soft thresholding power β was selected (Figures 11A, Figure 9) and three modules were obtained (Figures 11B,C). Red, blue, and green modules were positively correlated with the “high-immune infiltration” subgroup (clusters C and D) and negatively correlated with the “low-immune infiltration” subgroup (clusters A and B), *p* < 0.05 was used as the threshold. The turquoise module was positively correlated with clusters C and D and negatively correlated with cluster A. The brown module was positively correlated with cluster A and negatively correlated with clusters B and D. Pink and yellow modules were positively correlated with cluster B and negatively correlated with cluster A. In the blue module, which was most correlated with cluster D, the top ten hub genes selected by the MCC algorithm in the PPI network were CD28, CD8A, CSF2, GZMB, IFNG, IL15, IL2, IL2RA, IL7R, and PRF1(Figure 11D). Hub genes were positively correlated with most of the immune cells and immune functions, such as HLA, CCR, etc., (Figure 11E). A survival analysis showed that high expression of CD28, CD8A, IFNG, IL2RA, IL7R, IL15, and PRF1 had a poor prognosis whereas a better prognosis was found in IL2 and GZMB (Figure 12).

## Discussion and Conclusion

The prognosis of LGG patients had few significant improvements in the past 30 years. Individualized therapeutic schedules were because of the natural intrinsic heterogeneity of LGG (Deng et al., 2020). Li et al. (2021) provided a metabolic signature-based subgrouping method for LGG and Zhou et al. (2021) divided LGG into three clusters based on a tertiary lymphoid structure to provide potential treating options. Since the existence of an afferent system between the brain and peripheral immune system had been demonstrated (Qi et al., 2020), immunotherapy would be a promising strategy for its ability to penetrate the blood–brain barrier (Xu et al., 2020). Wu et al. (2020) classified diffuse LGGs into three immunological subtypes and found that the high lymphocytic and macrophage M2 infiltrate subtype indicated a worse prognosis. The immune microenvironment of LGG remained complicated. We studied the correlations between immunogenomic changes and immunity infiltration features in LGG to identify proper immune clusters and hub genes for precision treatment.

Our study identified four immune clusters in TCGA dataset and they were verified in two CGGA datasets. Cluster A, which had the lowest immune infiltration, was regarded as an “immune cold” phenotype. Clusters A and B could be considered a “low-immune infiltration” subgroup and both of them were closely correlated with “Immunologically Quiet” (C5) functional subtypes. Clusters C and D were considered the “high-immune infiltration” subgroup when cluster D was inferred as an “immune-rich” phenotype with the highest immune infiltration degree. In general, the expression of immune checkpoints such as PDCD1, CD274, PDCD1LG2, CTLA-4, LAG3, and HAVCR2 increased along with the order (cluster D > C > B > A), indicating higher hazards of immune escape in high-immune infiltration clusters. LGGs in clusters A and B tended to have a lower WHO grade, higher IDH1 mutation, and better overall survival than those in the “high-immune infiltration” subgroup. The LGGs in “immune-rich” cluster D showed significant PTEN and EGFR mutation frequencies and notable sensitivity to anti-immune checkpoint PD-1 therapy and the chemotherapy of cisplatin and gemcitabine. On the contrary, LGGs in the “low-immune infiltration” subgroup (clusters A and B) were more sensitive to bleomycin and doxorubicin. The results would provide potential individualized treatment recommendations for LGGs. Cluster D was enriched in KEGG pathways such as cytokine–cytokine receptor interaction, antigen processing and presentation, cell adhesion molecules (CAMs) and ECM-receptor interaction, reminding us that different immunophenotypes may be caused by changes in the aforementioned pathways. Meanwhile, RTK-RAS and TP53 pathways were affected in cluster D. In the blue module of WGCNA networks, CD28, CD8A, CSF2, GZMB, IFNG, IL15, IL2, IL2RA, IL7R, and PRF1 were selected as the hub genes which were closely correlated with most of the immune cells. Seven of them were correlated with a poor prognosis, two of them were protective prognostic factors and one of them had no significant association with prognosis.

An immune clustering analysis of our study indicated that high-immune infiltration would lead to a worse prognosis with immune checkpoint activation. This distinct feature might result from the immunocyte recoding by cytokines and chemokines in the LGG microenvironment (Hinshaw and Shevde, 2019). Immunocytes were turned into tumor-promoting phenotypes and conversely promoted tumor growth and immune evasion. In addition, the relatively lower IDH1 mutation and higher PTEN and EGFR mutation frequency in high immune infiltration clusters also supported the aforementioned inference. Although LGG patients in cluster D would suffer poor prognosis expectations, they might benefit from immune checkpoint PD-1 inhibitors and chemotherapeutic drugs of cisplatin and gemcitabine. Cisplatin and gemcitabine had shown encouraging tolerance and efficacy in clinical trials (Gertler et al., 2000; Massimino et al., 2002; Massimino et al., 2005; Hall et al., 2019). The TME in LGG appears to be different from other solid tumors because of the presence of the blood–brain barrier or properties of macrophages. In the present research, the M2-type macrophage was significantly enriched in primary LGG, and the proportions of macrophages can still constitute up to 50% in the TME of LGG. Some researchers demonstrated that high levels of M2-type macrophages were defined as the adverse prognostic factors in LGG. Conversely, high levels of M1-type macrophages and CD8+T cells were identified as protective factors. Apart from that, the revolution of drug delivery methods in nanoplatforms and liposomes had shown a promising future to precisely deliver the individualized chemotherapeutic drugs for LGGs (Shein et al., 2016; Renault-Mahieux et al., 2021; Wang et al., 2021). Most of the hub genes correlated with cluster D had the function of immunocyte activation. CD28 is involved in T-cell activation, cell proliferation induction, and T-cell survival. CD8 mediates efficient cell–cell interactions within the immune system. IFNG can activate effector immune cells and enhance antigen presentation. IL2RA is involved in the regulation of immune tolerance by controlling regulatory T cells. IL7R mediates the proliferation of lymphoid progenitors. IL15 stimulates the proliferation of T-lymphocytes. PRF1 plays a key role in defence against neoplastic cells. IL2 can stimulate B-cells, monocytes, lymphokine-activated killer cells, natural killer cells, and glioma cells. GZMB mediates target cell death and CSF2 promotes the production, differentiation, and function of granulocytes and macrophages. Although the majority of hub genes play a role in tumor promotion in the microenvironment of LGG, which was consistent with the poor prognosis expectation in cluster D, two hub genes termed IL2 and GZMB exerted a protective role in prognosis. It revealed the complex inherent interconnections of immunogenomic changes.

There are still some limitations in our study. First, we were unable to conduct an external validation in native cohorts. Second, we only used the ssGSEA and MCPcounter algorithms to corroborate our findings, and we will need to conduct assays to confirm our conclusion in the future. In conclusion, immunotyping of LGGs revealed the heterogeneity of the immune microenvironment and genomics changes. Our classifications would be beneficial for individualized prognostic prediction and anti-tumor therapy.

## Data Availability

The original contributions presented in this study are included in the article/Supplementary Material, further inquiries can be directed to the corresponding author.
