# Supplementary figures and images for: Identification of Immunological Characteristics and Immune Subtypes Based on Single-Sample Gene Set Enrichment Analysis Algorithm in Lower-Grade Glioma
Source: Front Genet. 2022 May 13;13:894865. doi: 10.3389/fgene.2022.894865 (PMC9136245; doi:10.3389/fgene.2022.894865)

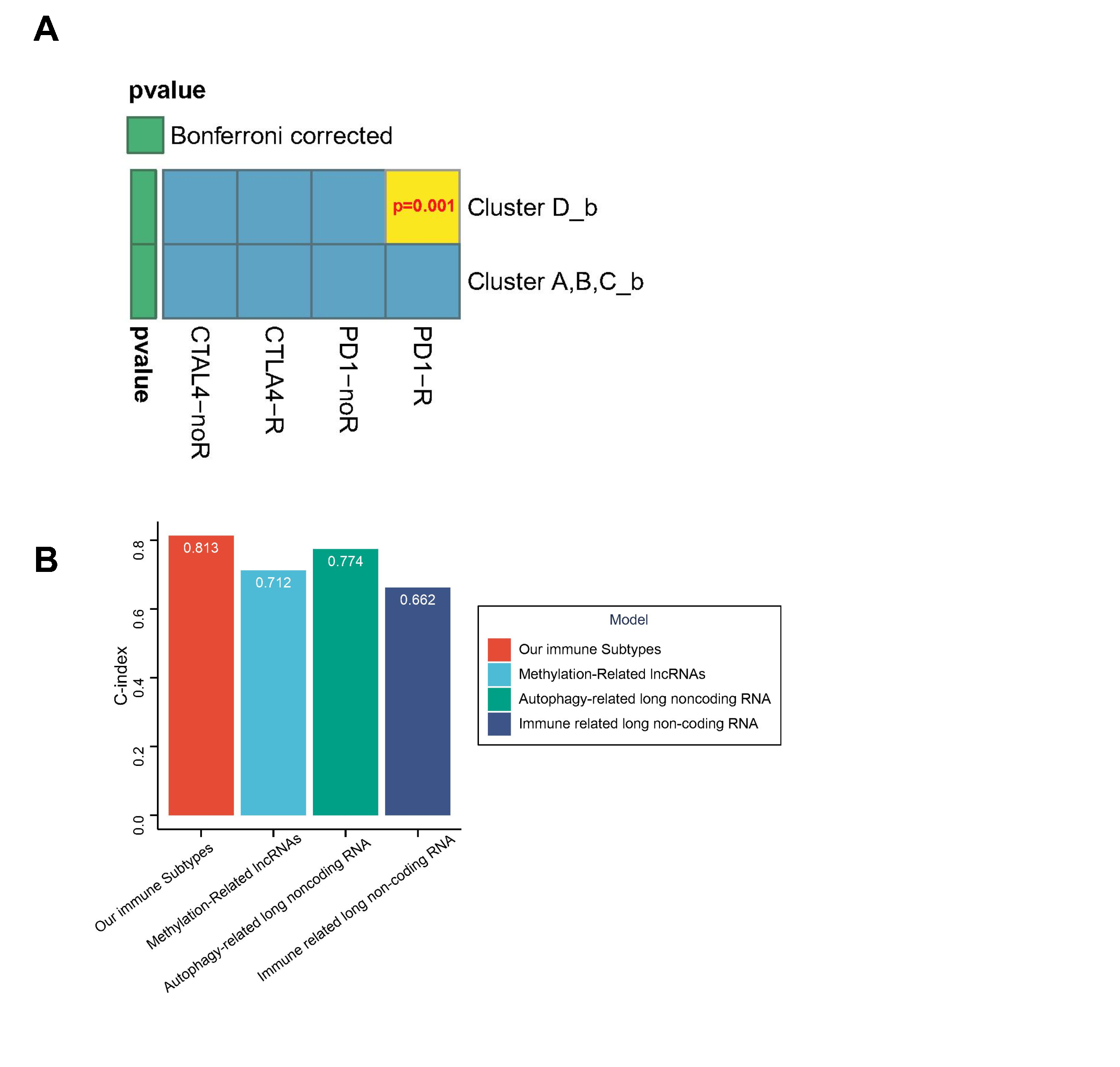

Supplement: Supplementary file 1 [file Image3.TIFF]

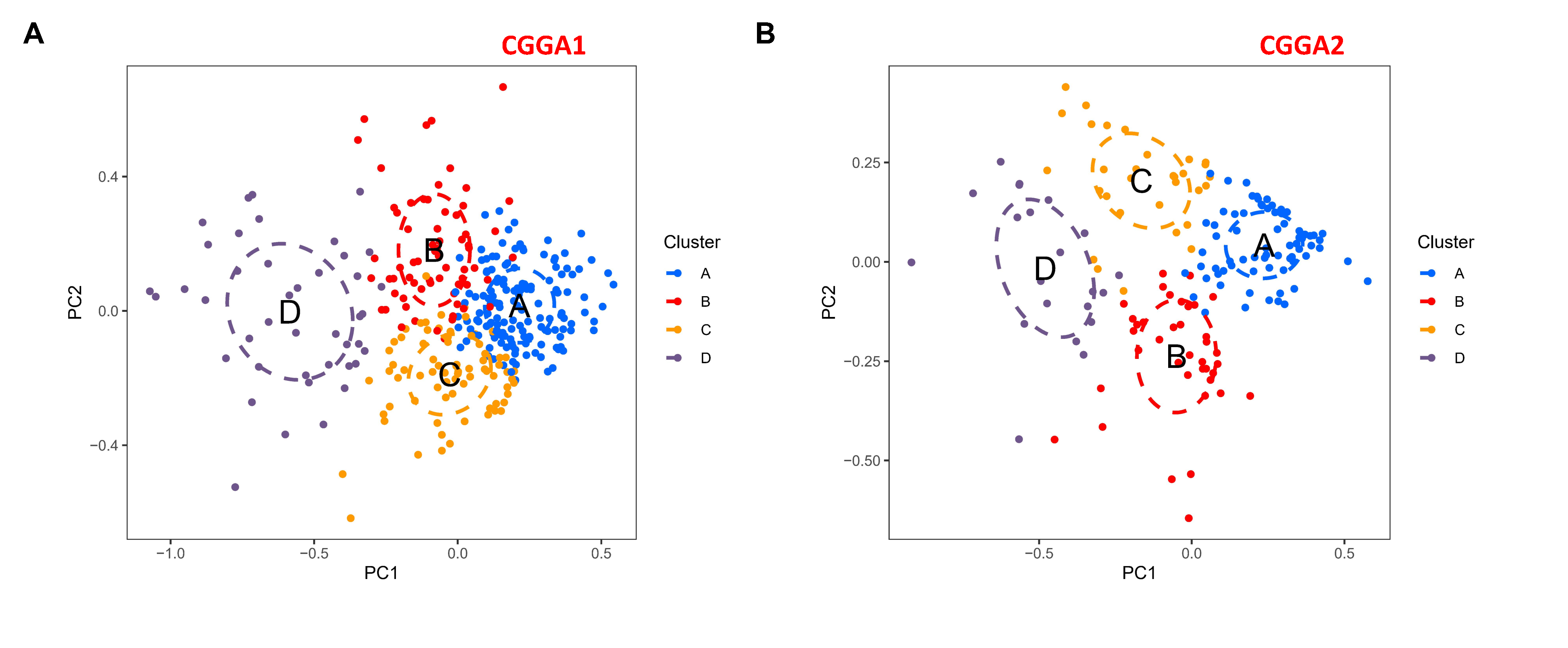

Supplement: Supplementary file 2 [file Image1.TIFF]

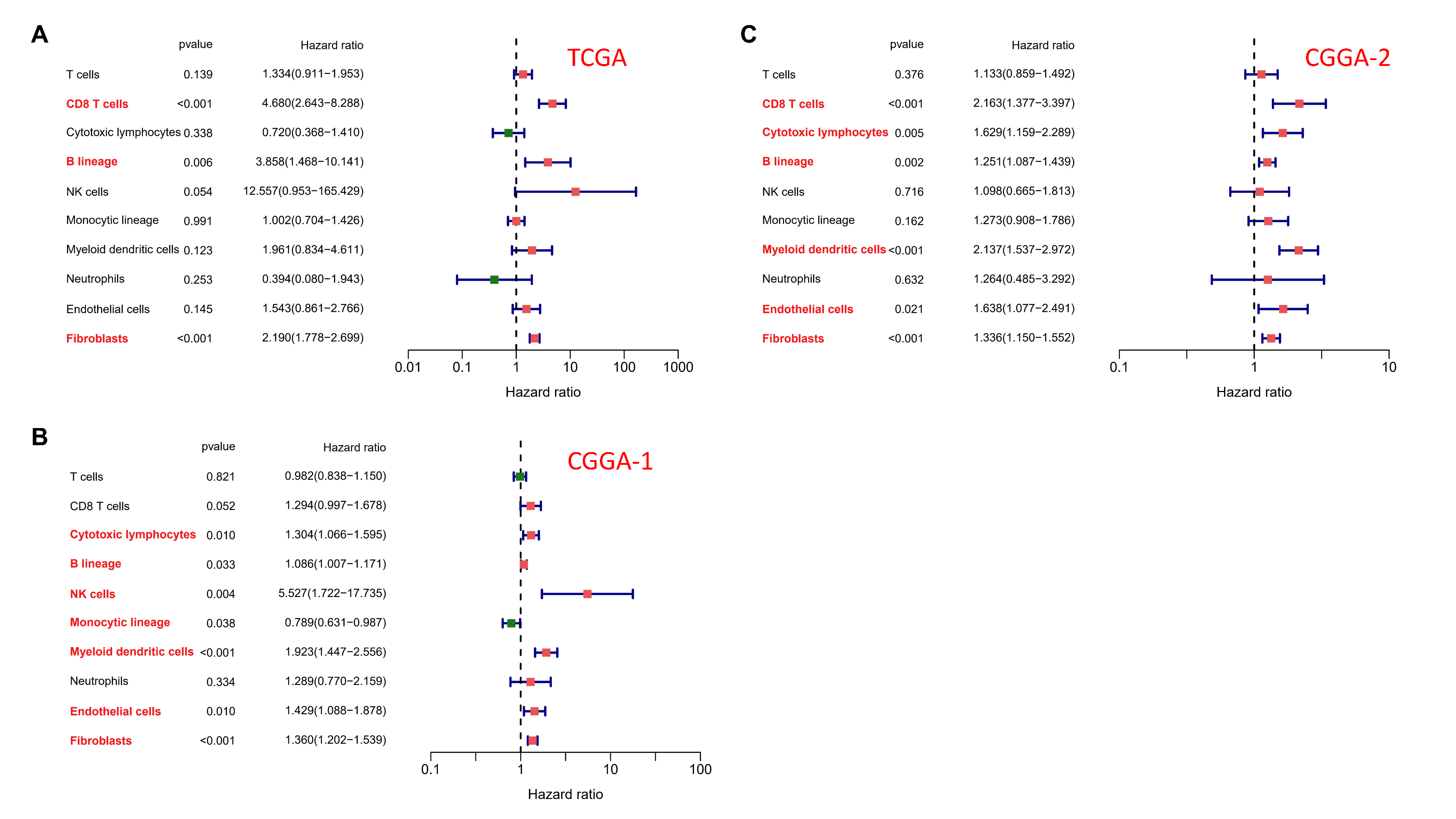

Supplement: Supplementary file 3 [file Image2.TIFF]
